# Preparation of Fly Ash/Epoxy Composites and Its Effects on Mechanical Properties

**DOI:** 10.3390/polym12010079

**Published:** 2020-01-02

**Authors:** Jeesoo Sim, Youngjeong Kang, Byung Joo Kim, Yong Ho Park, Young Cheol Lee

**Affiliations:** 1 Energy Plant Group, Korea Institute of Industrial Technology, Busan 46938, Korea; simexp95@kitech.re.kr (J.S.); yjkang@kitech.re.kr (Y.K.); kbj@kitech.re.kr (B.J.K.); 2 Department of Materials Science and Engineering, Pusan National University, Busan 46241, Korea

**Keywords:** epoxy, fly ash, viscosity, mechanical properties, fracture behavior

## Abstract

In this research, a fly ash/epoxy composite was fabricated using fly ash filler classified as industrial waste. The behavior of its mechanical properties was investigated by changing the volume of fly ash to 10, 30 and 50 vol.%. To determine the influence of particle size on the mechanical properties, we used two different sizes of the fly ash, which were separated by sieving to less than 90 μm and 53 μm. To optimize fabrication conditions, the viscosity of the fly ash/epoxy slurry was measured at various temperatures with different fly ash volume fractions. In terms of mechanical properties, tensile strength increased as the amount of fly ash increased, up to a critical point. On the other hand, the compression strength of the composite increased continuously as the amount of fly ash increased. Finally, the fracture surfaces were characterized and correlated with the mechanical properties.

## 1. Introduction

Recently, studies on energy saving due to environmental problems and exhaustion have been conducted worldwide. Composite properties are governed by combining several components into one material. Therefore, composites have resulted in a significant increase in specific strength, which has been applied to aircrafts, electronic components, automobiles, and sports equipment [1,2,3]. The mechanical properties of composites can be altered by the strength, shape, and volume fraction of the filler. Depending on the type of filler, the density of the material can be reduced and the strength and electrical properties can be increased [4,5,6].

One of the conditions for producing a uniform composite is to adjust the viscosity of slurry. In the composite, when the filler is not uniformly dispersed in the matrix and aggregates or precipitates, the predicted mechanical properties are not achieved. The solution to mechanical degradation is to adjust the slurry viscosity to produce polymer composites with uniformly dispersed fillers [7]. If the viscosity is high, a lot of mechanical energy is needed to mix the slurry; thus, mixing by hand is not easy. However, if the viscosity is low, the filler precipitates and a uniform composite is not produced. Therefore, as the amount of filler is changed, it is necessary to maintain the slurry at a constant viscosity to uniformly mix the composite by maintaining the slurry at the optimum viscosity.

In this study, the epoxy used as a matrix in the composites is the thermosetting resin most commonly used in the polymer matrix composite. Epoxy resins have the advantage of low shrinkage upon curing, good adhesion with other materials, and high strength. Therefore, epoxy is widely used in various industrial fields. Epoxy-based composites are made of inorganic [8,9,10,11] and organic [12,13] fillers. Research into polymer matrix composites has been conducted in various industrial fields to improve the strength and electrical properties—and to reduce weight—mainly through the addition of fibrous fillers, hollow sphere, and metal fillers. The fly ash used as filler in this study is a by-product, generated from the combustion of coal in thermal power plants. Fly ash is a collection of ashes flying into the atmosphere using a dust collector, and bottom ash is attached to the inner wall of the incinerator and drops to the bottom of the incinerator due to its own weight. Fly ash has the advantage of a low price and high strength, and several technologists have been interested in using of the fly ash as filler in polymer materials [14,15,16,17,18]. Investigations have been conducted on the tensile strength, impact strength change [19,20], and the mechanical properties of fly ash-fiber/epoxy composites [16]. The recycling of by-products from thermal power plants is considered useful for resource recycling and environmental protection.

Considering the effect of conditions such as viscosity and particle size on mechanical properties, this study fabricated composites using fly ash and epoxy. The mechanical properties of the composites were tested depending on the amount of fillers. In addition, the failure behavior of fly ash/epoxy composite was investigated by observing the microstructure of the fracture surface. 

## 2. Materials and Methods 

### 2.1. Materials

**Figure 1:**
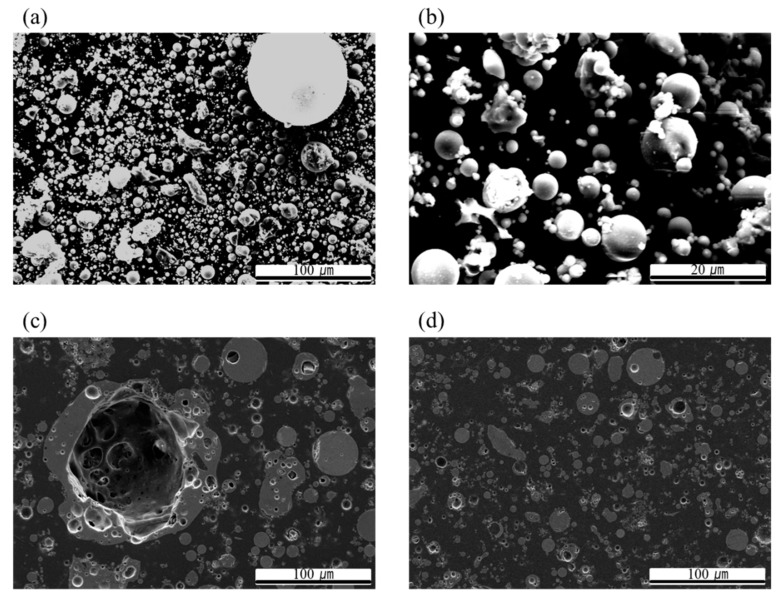
General microstructure under SEM for (**a**) Fly ash powder, low magnification, (**b**) Fly ash powder, high magnification, (**c**) Cross section of larger fly ash, (**d**) Cross section of smaller fly ash.

Diglycidyl ether of bisphenol A (Epoxy resin, epoxy equivalent weight (EEW, g/eq unit), 174 g/eq) was purchased from Dow Co. (Midland, MI, USA). The epoxy had a viscosity of 4–6 Pa∙s, a density of 1.16 g/cm^3^, and was mono-functional. Diluent (Mono-functional, EEW 275 g/eq) was purchased from Dow Co. Triethylenetetraamine (epoxy curing agent, amine hydrogen equivalent weight (g/eq unit), 24 g/eq) was purchased from Dow Co. The curing agent had a viscosity of 19.5–22.5 mPa∙s, and a density of 0.981 g/cm^3^. Gelling time was 16 h at 25 °C. The filler used as fly ash obtained from Hana Cement (Gunsan, Korea). The fly ash was a spherical or non-uniform shape. The density of the fly ash was 2.2 g/cm^3^. The main component of the fly ash was 54.3% of SiO_2_ and 25.8% of Al_2_O_3_. The detailed chemical composition of the fly ash is presented in Table 1. A particle size analyzer (Particle size analyzer, LS 13 320, Indianapolis, USA) was used to determine the particle size distribution of the fly ash, and fly ash powder was measured by the dry method using laser diffraction. The average particle size of the fly ash used in the experiment was 27 µm and the particle size distribution was 0.38–176.9 µm. To investigate the change in mechanical properties with particle size, sieving was conducted to less than 90 μm and less than 53 μm. As a result of the particle size analysis, the average particle sizes of less than 90 μm and less than 53 μm, were 18.7 μm and 17.3 μm, respectively. Confirming the shape of fly ash by Field emission scanning electron microscopy (FE-SEM; S-4800, HITACHI, Tokyo, Japan), found a similar size distribution to the particle size analysis result, but particles had various shapes (Figure 1). The surface of the ash during the sieving had similar results to the particle size analysis, but the shape of the fly ash varied. Most of the fly ash sieved to less than 53 μm was spherical (Figure 1b).

**Figure 2:**
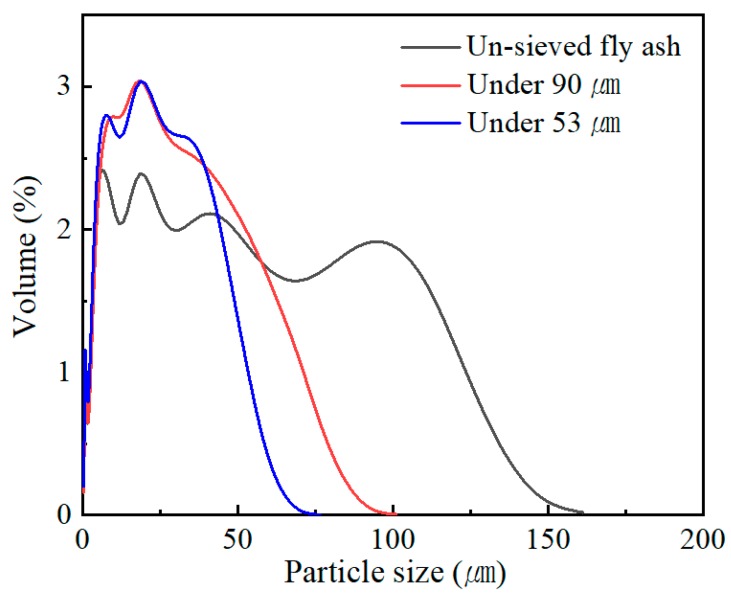
Particle size distribution of un-sieved fly ash, under 90 μm fly ash, under 53 μm fly ash.

A cross section of the fly ash is presented in Figure 2. The large particle fly ash of 80–90 μm is porous, but the fly ash of less than 53 μm is not. The fly ash less than 53 μm had an almost spherical shape. 

### 2.2. Characterization Methods for Resin Viscosity

The slurry was prepared by uniformly mixing the epoxy with a diluent of 5 wt.% relative to the mass of epoxy. First, the viscosity of the mixture was measured while increasing the amount of fly ash from 0 to 10, 30 and 50 vol.% at room temperature. Second, the change in viscosity with temperature was measured along with the viscosity change of slurry with 10, 30 and 50 vol.%. A viscometer (Viscometer; DV2T, BROOKFIELD, Middleborough, MA, USA) was used to measure the viscosity change rate, and the viscosity was measured using six kinds of spindles, R02, R03, R04, R05, R06, and R07. The slurry with low viscosity was measured with large radius R02-R04 spindle, and the slurry with high viscosity was measured with small radius R06 and R07.

### 2.3. Manufacture of Fly Ash/Epoxy Composite

Epoxy resin combined with a curing agent at a ratio of 100:14 (14 phr) by weight was used as matrix material. Diluents were mixed at a 5 wt.% weight ratio of epoxy resin, used as produced smooth production and de-molding. The resin was placed in an oven for 30 min in order to optimize the viscosity. Before curing, the weighed fly ash was added to the liquid matrix and the mixture was stirred with overhead stirrer for 10 min or more. To compare the mechanical properties depending on particle size, composites were prepared by sieving less than 90 μm and 53 μm fly ash, respectively. To minimize entrapped air in the composite, the mixture was cured by applying a pressure of 4 bar in the autoclave. In order to completely cure the polymer composite, it cured at autoclave for one day. Post curing of the specimen was performed for 1 hour at 100 °C, and then three to four days at room temperature. A compression test specimen was produced through a cylindrical mold 12.7 Φ*25.4 mm. The tensile test specimen was produced in a dog-bone like shape followed by standard ASTM D 638.

### 2.4. Mechanical Properties

The upper and lower parts of the compressed specimens were worked in parallel, and the mass-to-volume ratio was calculated depending on ASTM D 792. A tensile test was carried out by the ASTM D 638 test method, using a universal testing machine (AG-X plus 50 kN, Shimazu, Japan) with the deformation rate set to 5 mm/min. A compression test was conducted at a test speed of 5 mm/min by the ASTM D 695 test method using the same universal testing machine as the tensile test. At least five specimens were tested in each test, and the average value used. FE-SEM was used to observe fracture surfaces of tensile specimens.

## 3. Results

### 3.1. Effect of Viscosity

#### 3.1.1. Viscosity of Composites at Various Temperatures

**Figure 3:**
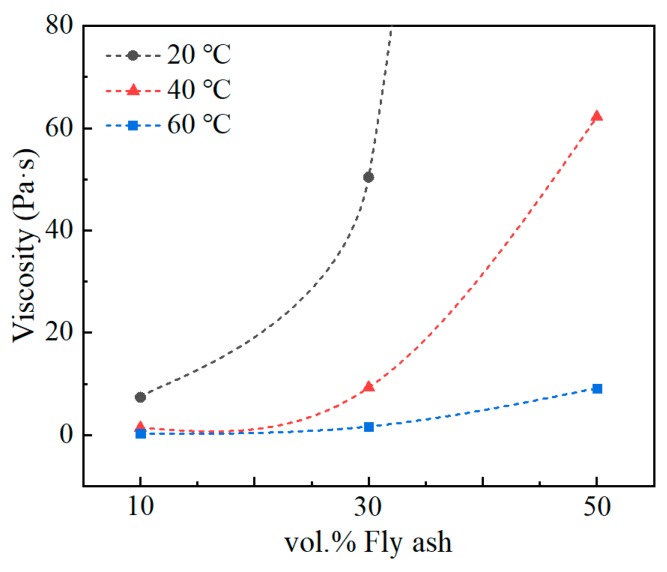
Viscosity distribution with fly ash content for composites at different temperature: (●) 20 °C, (▲) 40 °C and (■) 60 °C.

In general, the viscosity of composite is predominantly influenced by the type and amount of filler, resin matrix. In this study, since the kinetics of the dispersion system of fine particles in solution depended on the hydrodynamic forces acting on the surface of the particles, the viscosity was analyzed as the various filler content increased with respect to the vol.% rather than wt.% [21]. The viscosity measurement results, depending on different volume fraction and temperature are shown in Figure 3. Viscosity measurements of less than 90 µm and 53 µm fly ash showed similar values, because two kinds of fly ash had a similar average particle size (18.7 μm and 17.3 μm). As the temperature increases, the viscosity of the slurry decreases as the fluidity of the epoxy increases, viscosity change results depending on filler volume fraction and temperature have already been studied [22]. Furthermore, the viscosity of the slurry decreased with an increasing temperature at the equivalent fly ash volume fraction. The viscosity result possible to fit exponential graph shape was shown in the following Equation (1) [7]:

(1) $$y=Ae^{\mathrm{bx}}$$

The A and b coefficients of the function are calculated from Figure 3, and presented in Table 2. The A value decreases as the temperature increases compared to the same volume fraction increase. In addition, coefficient b was confirmed to have a similar value regardless of the increase in temperature. To explain this aspect, the function calculated as above can be explained in connection with Mooney’s equation. This Equation (2) was formulated for changing the viscosity of composites by the volume content of filler and resin viscosity [23].

(2) $${ŋ}_{C}={ŋ}_{R}\exp\left(\frac{2.5ø}{1-kø}\right)$$

ŋ_c_ = viscosity of composite, ŋ_R_ = resin viscosity, ø = filler volume fraction (0 < ø <1), κ = self-crowding factor. Matching Equation (1) to (2), demonstrates that the calculated constant A and b is consistent with ŋ_R_ and (2.5/(1 – k)), respectively. As the temperature decreased from 60 °C, to 40 °C, to 20 °C, the viscosity of resin increased by six and twenty times, respectively. In addition, constant b showed that the self-crowding factor had a similar value regardless of temperature because the added kind of fly ash was the same. Therefore, in the same shape as Mooney’s equation, the viscosity of the composite increases exponentially as the volume fraction of the filler increases (Figure 3, Table 3).

#### 3.1.2. Mechanical Properties of Composites According to Viscosity

**Figure 4:**
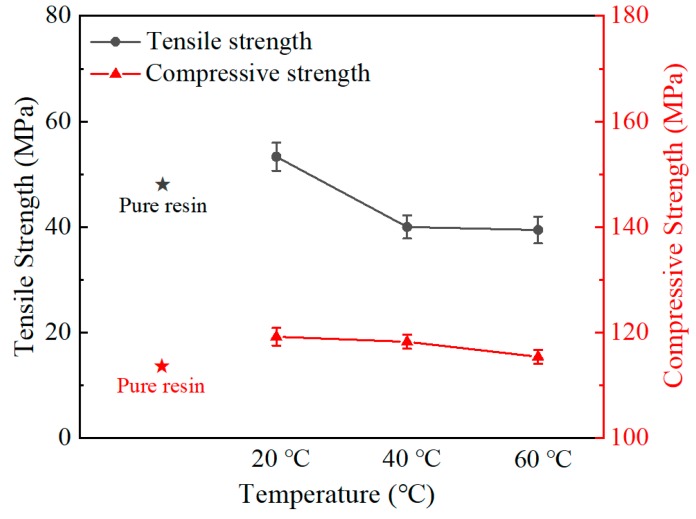
Tensile and Compressive strength of 10 vol.% fly ash/epoxy composite prepared at 20 °C, 40 °C, and 60 °C.

Figure 4 presents the tensile and compression test results of 10 vol.% fly ash/epoxy composite produced at 20 °C, 40 °C and 60 °C. The mechanical properties of 10 vol.% fly ash composite, which was produced at 7.3, 1.4, and 0.3 Pa∙s at 20 °C, 40 °C, and 60 °C, were measured. When fabricated under low viscosity, tensile strength was reduced by 25% (53.3→39.4 MPa). Compressive strength was also reduced by 3% (119.2→115.4 MPa). At low viscosity, layer separation due to density difference easily occurs [24] (40 °C, 60 °C). The higher viscosity of slurry has high resistance to preventing the filler from settling because of low fluidity of the liquid phase (20 °C). The slurry with the optimum viscosity, which has a slow settling rate, is cured in a uniformly dispersed state of the filler, and results in higher strength. The mechanical properties of the 10 vol.% fly ash/epoxy composites, according to the curing conditions, confirmed that the optimum viscosity was about 7–9 Pa∙s at 20 °C.

Table 4 presents the mechanical properties of the composites fabricated with various viscosities and volume fractions. When fabricating composites under different temperatures, the optimum mechanical properties were different depending on the amount of fly ash added. The composites produced at the fly ash 10 vol.%—20 °C, 30 vol.%—40 °C, and 50 vol.%—60 °C conditions showed the highest mechanical properties. Through these results, the manufacturing viscosity condition with higher strength were 7–9 Pa∙s. The reason for the increase in temperature at a higher volume fraction is that in high viscosity conditions, the slurry cannot be mixed by mechanical mixing. This is because as the content of the filler added to the matrix increases, the torque applied to the spin results in a high viscosity as indicated. In addition, a large number of pores are generated in the composite, so a uniformly dispersed material is not produced [25]. Mixtures of high viscosity/high content fly ash cause defects such as pores, and low viscosity/low content fly ash cause filler precipitation. Because of this phenomenon, the manufactured composite had low mechanical properties. Therefore, the composites were maintained at optimal condition of all filler volume fractions for the uniform dispersion of fly ash.

### 3.2. Effect of Filler Size

#### 3.2.1. Density

**Figure 5:**
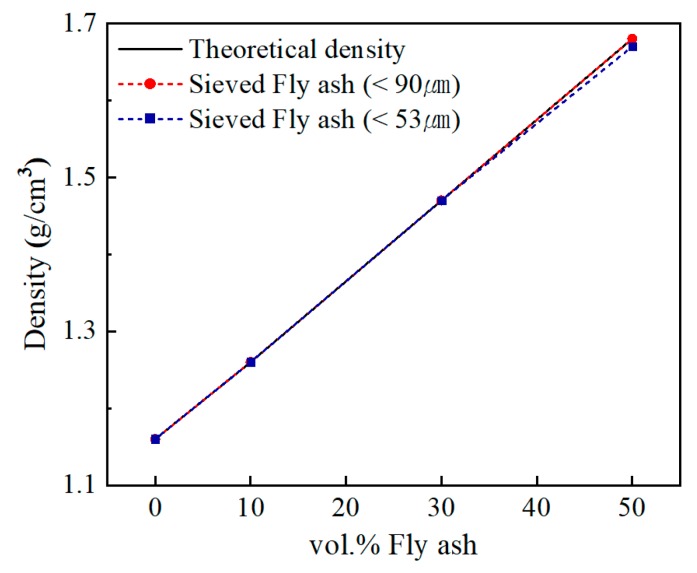
Density variation with fly ash content for composite containing: (●) less than 90 μm, (■) less than 53 μm.

Figure 5 shows the density of composites with increasing fly ash content. If there was no defect in the composite, the experimental density of the fly ash and epoxy was calculated using the Archimedes principle. The mixing equation for calculating the density of the composite was calculated using rule-of-mixture by the following Equation (3).

(3) $$\rho_{t}=\;\frac{1}{\left(\frac{W_{m}}{\rho_{m}}\right)+\left(\frac{W_{p}}{\rho_{p}}\right)}$$

*W* and *ρ* represent weight fraction and density, respectively. Subscript lowercase letters *m*, *p*, and *t* represent matrix, filler, and composite, respectively. When the strength of the filler added in the composite was weak, the filler breaks during the mixing process and pores were generated in the composite. The breakage and pore generation of the filler is a defect, resulting in a difference between the theoretical density and the experimental density. However, the added fly ash was strong, and no filler breakage was expected. Since the manufacturing conditions were optimized by adjusting the viscosity of the composite, the two values did not differ greatly. The density increased by about 8.6% with each increase of 10 vol.% of fly ash. This is because the density of the composite increased in proportion to the inverse of the fly ash density of 2.2 g/cm^3^. As the fly ash content increased by 10, 30, and 50 vol.%, the density increased to 8.6%, 26.7%, and 44.8%. 

#### 3.2.2. Tensile Test

**Figure 6:**
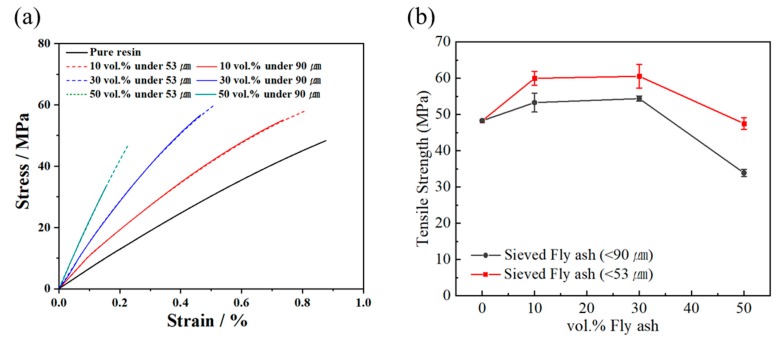
(**a**) Tensile stress–strain curves of composite containing less than 90 μm, 53 μm fly ash, (**b**) Tensile strength of the fly ash/epoxy composite.

Figure 6a is a graph of the tensile properties of fly ash/epoxy composites depending on the fly ash volume fraction and size. Figure 6b is a graph of the tensile strength of the fly ash/epoxy composites. The strength of the epoxy matrix was 48 MPa, and the elongation was 0.8%. The epoxy matrix exhibits brittle properties without plastic deformation zones. Fly ash is also considered a brittle material [26]. The brittle matrix-filler composites have higher strength—but lower elongation—as filler content increases. When the size distribution of the fly ash particles was reduced from less than 90 μm to less than 53 μm, the initial tensile graphs were very similar. However, under 90 μm fly ash composite break more quickly. When 50 vol.% fly ash was added, under 53 μm fly ash filled epoxy were destroyed at 0.2% elongation.

Regardless of particle size distribution, tensile strength was increased compared to pure resin when 10 vol.% and 30 vol.% fly ash were added. Increasing the volume fraction of the fly ash, composites were strengthening because strength of fly ash was higher than resin matrix. However, as the fly ash volume fraction increased, the interface of fly ash and resin caused many defects (e.g., the de-bonding of filler and resin). In this experiment, fly ash/epoxy composites had a maximum tensile strength value at 30 vol.%.

**Figure 7:**
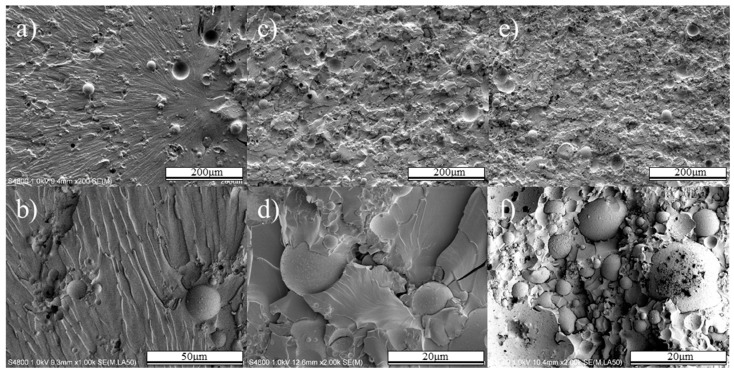
Fracture surface of less than 53 μm fly ash/epoxy composite in the direction of tensile loading. 10 vol.% fly ash/epoxy composite ((**a**) low magnification, (**b**) high magnification), 30 vol.% fly ash/epoxy composite((**c**) low magnification, (**d**) high magnification), 50 vol.% fly ash/epoxy composite ((**e**) low magnification, (**f**) high magnification.).

**Figure 8:**
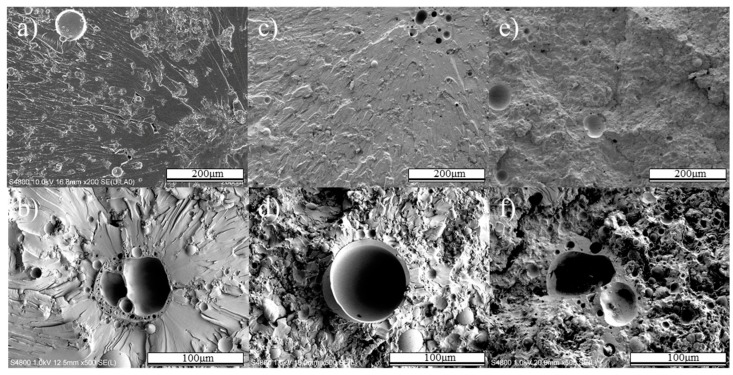
Fracture surface of less than 90 μm fly ash/epoxy composite in the direction of tensile loading. 10 vol.% fly ash/epoxy composite ((**a**) low magnification, (**b**) high magnification), 30 vol.% fly ash/epoxy composite ((**c**) low magnification, (**d**) high magnification), 50 vol.% fly ash/epoxy composite ((**e**) low magnification, (**f**) high magnification.).

The fracture surface morphologies of epoxy and composites are shown in Figure 7 and Figure 8. Cleavage features of the epoxy matrix were observed at the fracture surface (in Figure 7) of composites with 10 vol.% fly ash Under 53 μm size. The fly ash and crater were observed in fracture SEM images. The crater shape is a round sphere, like a fly ash. The fly ash existed between the failure initiation region and the cleavage feature line. This phenomenon indicates that the de-bonding caused by the poor interfacial bonding between epoxy and fly ash [27]. In the 10 vol.% fly ash composite, the matrix had a greater influence on the tensile properties than the fly ash. A shorter cleavage feature was observed at the matrix of the fracture surface (Figure 7c) when the fly ash content increased to 30 vol.%. When 50 vol.% fly ash was added, the cleavage feature was shorter again and the fly ash was present at the site of failure (Figure 7e,f). When the fly ash content was more than 50 vol.%, the de-bonding of the fly ash and epoxy intensified, resulting in a 27.7% decrease in the strength of the composite and a 30.3% decrease in the elongation.

Epoxy resin displayed similar changes as the volume fraction increased. However, large-size fly ash was observed with broken surface. As demonstrated in Figure 8, the diameter of the broken fly ash was more than 53 μm and there were many pores inside. The thin walls of the fly ash with pores inside are susceptible to mechanical forces and are prone to breakage. Thus, composites with large particles added have poorer mechanical properties than composites with only small particles.

#### 3.2.3. Compression Test

**Figure 9:**
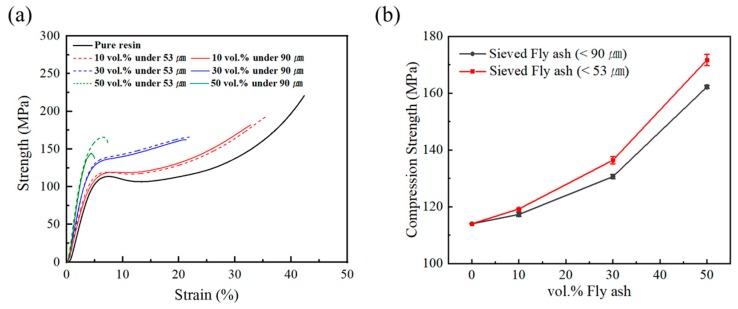
(**a**) Compressive stress–strain curves of composite containing less than 90 μm, less than 53 μm fly ash, (**b**) Compression strength of the fly ash/epoxy composite.

Figure 9a is a graph of the compression characteristics of fly ash/epoxy composites with less than 90 µm fly ash in different volume fraction. The compressive yield strength of pure epoxy is 114 MPa, and the compression strength of 10 vol.% fly ash/epoxy composite is 119 MPa. Unlike the brittle tensile properties, the plastic deformation of compressive properties occurs after the yield point. After reaching the compressive yield strength, pure resin showed buckle behavior in which stress degradation occurs. This phenomenon is due to permanent deformation of the polymer chains [27]. As shown in Figure 9b, the compressive yield strength of composite with fly ash less than 53 µm increased by 10% (114→119→136→171 MPa) in the case of composites with fly ash filled at 0~50 vol.%. As the volume fraction increases, the plastic deformation zone decreased due to the brittle fly ash addition. It is noted in Figure 9a, that the stress drop region did not occur after the yield point in the 30 vol.% fly ash composite, and plastic deformation did not occur when 50 vol.% fly ash was added. As the amount of fly ash increased, the effect of fly ash on the composites also increased, and the compression behavior changed from ductile to brittle. Compared with the various particle sizes of the fly ash/epoxy composite, the mechanical properties of the composites decreased slightly at less than 90 μm fly ash composite. Furthermore, as the fly ash content was increasing, the difference in strength between less than 90 μm and 53 μm was more pronounced. This phenomenon is caused by destruction from thin walls caused by multi pores inside large fly ash particles.

Based on the fracture mechanisms of a typical ductile matrix-brittle reinforced filler composite system [28], the initial compressive stress is shared between the matrix and the filler. However, when a relatively low strength matrix crack initiates, the filler acts as crack stopper and provides increasing fracture resistance to mechanical loads. The higher the filler content, the greater the fracture resistance to these external forces. Therefore, the composites had greater strength than the pure resin, but the elongation of the composite decreased as the ductile matrix volume fraction decreased. Compared with the tensile properties of brittle filler-brittle matrix properties, the compressive properties of brittle filler-ductile matrix properties were strengthened (Figure 9b).

## 4. Conclusions

In this study, the mechanical properties of composite were tested by changing particle size, volume fraction and temperature. The mechanical properties of the composites were improved by optimizing the fabrication conditions by controlling the viscosity of the composites. The main conclusions of this study are as follows:

(1) During the manufacturing of the composite, viscosity conditions were optimized by controlling the temperature of the slurry (mixing resin and filler). The mechanical properties of the composites produced at a viscosity of 7–9 Pa∙s performed best. As the filler content increased, the optimum manufacturing temperature also increased (fly ash 10 vol.%—20 °C, 30 vol.%—40 °C, and 50 vol.%—60 °C). By the optimum viscosity established, we predict that if the composite is manufactured with any other filler contents, the mechanical properties can be improved by manufacturing at the optimized viscosity condition.

(2) The mechanical properties of composite were tested containing fly ash less than 90 μm and 53 μm with 10, 30 and 50 vol.%. Tensile strength increased as the amount of fly ash increased up to a critical threshold, and then decreased with greater additions. The fracture behavior of tensile specimens showed the weak filler/matrix interface bonding and correlated with tensile properties. Therefore, the result of optimum fly ash content to improve the tensile properties were 30 vol.%. The strength of the compressive properties increased continuously. The increase in compressive properties appears to be due to the difference in the tensile-compressive properties of the epoxy matrix. 

(3) In addition, due to the pores present in the fly ash particles larger than 50 μm, the mechanical properties of the composite containing less than 90 um fly ash was reduced. Hollow-shaped large fillers such as fly ash cause significant decreases in strength, due to the thinner and weaker walls of the particles. It is concluded that composites with smaller particle size fly ashes showed significant improvement in mechanical properties.

## Figures and Tables

**Table 1:** Chemical composition of fly ash (unit: wt.%).

SiO_2_	Al_2_O_3_	Fe_2_O_3_	CaO	MgO	K_2_O	SO_3_	Na_2_O
54.3	25.8	7.41	5.69	1.51	1.43	0.66	0.5

**Table 2:** Regression analysis of the composite viscosity of the experimental composites as a functional of temperature. ($y=e^{\mathrm{bx}}$).

Temperature	A	b	R Statistic
20 °C	3.41	8.84	0.9966
40 °C	0.649	9.07	0.9992
60 °C	0.107	8.93	0.9994

**Table 3:** Experimental results of fly ash filled epoxy composite at different temperature.

Volume (%)	Viscosity at 20 °C (Pa∙s)	Viscosity at 40 °C (Pa∙s)	Viscosity at 60 °C (Pa∙s)
0	3.69	0.456	0.09
10	7.36	1.41	0.256
30	39.8	8.53	1.66
50	681	62.2	8.9

**Table 4:** Mechanical properties of fly ash filled epoxy composite at different temperature.

Fly Ash Volume Fraction (%)	Temperature (°C)	Viscosity (Pa∙s)	Tensile Strength (MPa)	Compressive Strength (MPa)
10	20	7.36	53.3	119.2
10	40	1.41	40	118.3
10	60	0.26	39.4	115.4
30	20	39.8	None	None
30	40	8.53	53.3	136.5
30	60	1.65	42.5	125.7
50	20	681	None	None
50	40	62.2	None	None
50	60	8.9	33.9	165.6
